# Supplementary material for: Remodeling of gene regulatory networks underlying thermogenic stimuli-induced adipose beiging
Source: Commun Biol. 2022 Jun 14;5:584. doi: 10.1038/s42003-022-03531-5 (PMC9197980; doi:10.1038/s42003-022-03531-5)
Supplement: Supplementary file 3 — Reporting Summary [file 42003_2022_3531_MOESM3_ESM.pdf]

nature portfolio

Corresponding author(s): Paul D. Soloway

Last updated by author(s): Mar 27, 2022

Reporting Summary

Nature Portfolio wishes to improve the reproducibility of the work that we publish. This form provides structure for consistency and transparency in reporting. For further information on Nature Portfolio policies, see our Editorial Policies and the Editorial Policy Checklist.

Statistics

For all statistical analyses, confirm that the following items are present in the figure legend, table legend, main text, or Methods section.

n/a	Confirmed	
☐	☒	The exact sample size (*n*) for each experimental group/condition, given as a discrete number and unit of measurement
☐	☒	A statement on whether measurements were taken from distinct samples or whether the same sample was measured repeatedly
☐	☒	The statistical test(s) used AND whether they are one- or two-sided<br>*Only common tests should be described solely by name; describe more complex techniques in the Methods section.*
☒	☐	A description of all covariates tested
☐	☒	A description of any assumptions or corrections, such as tests of normality and adjustment for multiple comparisons
☐	☒	A full description of the statistical parameters including central tendency (e.g. means) or other basic estimates (e.g. regression coefficient) AND variation (e.g. standard deviation) or associated estimates of uncertainty (e.g. confidence intervals)
☐	☒	For null hypothesis testing, the test statistic (e.g. *F*, *t*, *r*) with confidence intervals, effect sizes, degrees of freedom and *P* value noted<br>*Give P values as exact values whenever suitable.*
☒	☐	For Bayesian analysis, information on the choice of priors and Markov chain Monte Carlo settings
☒	☐	For hierarchical and complex designs, identification of the appropriate level for tests and full reporting of outcomes
☐	☒	Estimates of effect sizes (e.g. Cohen's *d*, Pearson's *r*), indicating how they were calculated

Our web collection on statistics for biologists contains articles on many of the points above.

Software and code

Policy information about availability of computer code

Data collection	No computer code was used to collect the data.
Data analysis	Fastq files from two sequencing runs were merged and demultiplexed by adding the cell barcode to each read. Demultiplexed pair-end reads were aligned to mm10 using Bowtie2 (v2.4.3) with the parameters: bowtie2 -p 16 -t -X 2000-no-mixed-no-discordant. After alignment, reads were sorted by read name using samtools (v.1.11). Pair-end reads with low mapping quality fragments < MAPQ 30 and improperly paired fragments (SAM flag = 1804) were removed. Reads were separated based on the cell barcode and reads belonging to the same cell barcode were deduplicated. PCR duplicate reads identified by Picard (v2.26.1) 'MarkDuplicates' and the reads aligned to mitochondrial genome were removed. For initial filtering, we filtered out cells which have less than 1,000 reads. Then we first generated a cell-by-10kb-window sparse matrix using snapATAC. Then, we used Signac (v.1.1.1) for further analysis. After initial clustering, all cells in individual clusters were aggregated. For each cluster, peak calling was performed using the MACS2 (v2.1.4) 'callpeak' command with the parameters: -nomodel-shift -100-extsize 200-keep-dup-all -q0.05. The summits were extended ±250bp and the peak sites overlapping with the ENCODE mm10 blacklist were filtered. The peak sites identified from each cluster were merged and used to create a cell-by-peak matrix. To create a gene activity matrix, we counted the reads on the gene coordinates for the mouse genome from EnsembleDB extended to include the 2kb upstream. Doublet identification was performed using rScrublet R package. Differential abundance testing was performed using MiloR (v.0.99.6) R package. Gene Ontology analysis and KEGG pathway enrichment analysis of genes were carried out by clusterProfiler (v3.14.3). Biological function analysis of peaks was carried out by rGREAT (v1.22.0) R package. Motif analysis and co-accessibility were performed using chromVAR (v1.8.0) and Cicero (v1.3.4) respectively. Cell-cell interaction ligand-receptor analysis was performed using CellChat (v1.0.0) R package. Pseudotime trajectory analysis was carried out by ArchR (v1.0.1) R package. All codes were run using R (v3.5.0) and can be available upon request.

For manuscripts utilizing custom algorithms or software that are central to the research but not yet described in published literature, software must be made available to editors and reviewers. We strongly encourage code deposition in a community repository (e.g. GitHub). See the Nature Portfolio guidelines for submitting code & software for further information.

Data

Policy information about availability of data

All manuscripts must include a data availability statement. This statement should provide the following information, where applicable:

- Accession codes, unique identifiers, or web links for publicly available datasets
- A description of any restrictions on data availability
- For clinical datasets or third party data, please ensure that the statement adheres to our policy

snATAC-seq data reported in this paper is deposited in the NCBI Gene Expression Omnibus under accession number GSE185377. The GEO accession number for the scRNA-seq and snRNA-seq data is GSE133486. Previously published mouse brown adipose tissue PGC-1α ChIP-seq data from GSE110056. Publicly available mouse liver ESRRα ChIP-seq data used in our analysis was obtained from GSE43638. Mouse skeletal myoblasts MEF2α ChIP-exo data was obtained from GSE61207. H3K27ac and H3K4me1 ChIP-seq data from beige adipocytes after cold exposure and white adipocytes at 30°C were obtained from GSE108077.

Field-specific reporting

Please select the one below that is the best fit for your research. If you are not sure, read the appropriate sections before making your selection.

☒ Life sciences ☐ Behavioural & social sciences ☐ Ecological, evolutionary & environmental sciences

For a reference copy of the document with all sections, see nature.com/documents/nr-reporting-summary-flat.pdf

Life sciences study design

All studies must disclose on these points even when the disclosure is negative.

Sample size	Three biological replicates (three mice) for each group were used for snATAC-seq libraries.
Data exclusions	Reads were excluded if indexes did not match predetermined barcode sequences. Barcodes were filtered as described in the manuscript.
Replication	All three batches of snATAC-seq experiments were performed independently.
Randomization	Mice were randomly assigned to cold (6.5°C) or CL-316,243 (CL, 1 mg/kg), a β3-adrenergic receptor (Adrb3) selective agonist treatment group.
Blinding	Blinding is not relevant to our study, as our statistical tools are not dependent on blinding.

Reporting for specific materials, systems and methods

We require information from authors about some types of materials, experimental systems and methods used in many studies. Here, indicate whether each material, system or method listed is relevant to your study. If you are not sure if a list item applies to your research, read the appropriate section before selecting a response.

Materials & experimental systems

n/a	Involved in the study	
☒	☐	Antibodies
☒	☐	Eukaryotic cell lines
☒	☐	Palaeontology and archaeology
☐	☒	Animals and other organisms
☒	☐	Human research participants
☒	☐	Clinical data
☒	☐	Dual use research of concern

Methods

n/a	Involved in the study	
☒	☐	ChIP-seq
☒	☐	Flow cytometry
☒	☐	MRI-based neuroimaging

Animals and other organisms

Policy information about studies involving animals; ARRIVE guidelines recommended for reporting animal research

Laboratory animals	C57BL/6J 6-weeks-old male mice were purchased from Jackson Laboratories.
Wild animals	This study did not involve wild animals.
Field-collected samples	This study did not involve field-collected samples.

Ethics oversight	The experiments were performed with permission of the Institutional Animal Care and Use Committee (IACUC) at Cornell University (Protocol number 2017-0063).

Note that full information on the approval of the study protocol must also be provided in the manuscript.
